# Spray Analysis of Biodiesels Derived from Various Biomass Resources in a Constant Volume Spray Chamber

**DOI:** 10.1021/acsomega.2c00952

**Published:** 2022-06-02

**Authors:** Anılcan Ulu, Güray Yildiz, Alvaro Diez Rodriguez, Ünver Özkol

**Affiliations:** † Izmir Institute of Technology, Faculty of Engineering, Department of Mechanical Engineering, Urla, 35430 Izmir, Turkey; ‡ Izmir Institute of Technology, Faculty of Engineering, Department of Energy Systems Engineering, Urla, 35430 Izmir, Turkey

## Abstract

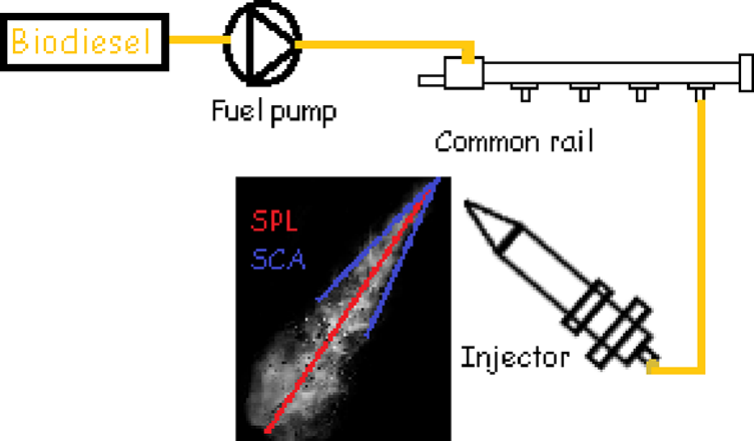

This research aimed to analyze the spray characteristics of various biodiesels, which have rarely been investigated in terms of spray analysis in the literature compared to fossil diesel. For this purpose, four different methyl ester-type biodiesels were produced from canola, corn, cottonseed, and sunflower oils. These feedstocks were selected due to their wide availability in Turkey and being among the significant resources for biodiesel production. Measured physical properties of biodiesel samples showed that biodiesel fuels had, on average, 1.7 to 1.9 times higher viscosities, 5.3 to 6.6% larger densities, and 37 to 39.1% higher contact angle values than the reference diesel fuel. Spray characteristics of all fuels were experimentally examined in a constant volume spray chamber under chamber pressures of 0, 5, 10, and 15 bar and injection pressures of 600, 800, and 1000 bar. All tested biodiesels performed, on average, 3 to 20% longer spray penetration lengths, 5 to 30% narrower spray cone angles, and 5–18% lesser spray areas than the reference diesel fuel under chamber pressures of 5 and 10 bar. No significant differences occurred at 15 bar ambient pressure between biodiesels and diesel. In addition, analytical and empirical predictions showed that biodiesels had around 21.2–35.1% larger SMD values and approximately 7% lower air entrainment.

## 1 Introduction

Diesel engines have been leading devices for power generation in various sectors (e.g., road, rail, maritime transportation, agriculture), and they still maintain their importance in power generation today. For example, in 2019, around 4.65 million passenger cars having diesel engines were produced in the European Union (EU).^1^ On the other hand, diesel engines have been the focus of criticism for a long time due to their pollutant emissions. The EU and the Environmental Protection Agency (EPA) in the United States of America (USA) brought strict regulations on pollutant levels from motor vehicles.^2^ For instance, in 2011, the EU introduced Euro 6 vehicle emission standards,^3^ which still apply to today’s cars. According to these standards, light-duty vehicles having diesel engines can emit at most 500 mg of carbon monoxide (CO), 80 mg of nitric oxides (NO_*x*_), a total value of 170 mg of NO_*x*_ and hydrocarbons (HC), and 5 mg of particulate matters (PM) per kilometer.^4^ Diesel fuel consumption is still high around the globe, although their utilization in diesel engines is harmful to the environment. In 2019, ca. 3746 thousand tonnes of diesel fuel were globally utilized daily, and Europe consumed approximately 23.8% of these.^5^

Alternative types of drop-in fuels need to be used for a purpose of decreasing fossil fuel usage.^6^ One promising alternative to conventional diesel fuels is biodiesel. In 2019, the global biodiesel consumption was around 104 thousand tonnes per day, and European countries used approximately 39.8% of the total biodiesel.^5^ Biodiesels can be ideal sources for power generation in diesel engines owing to the following advantages: having higher flash points resulting in ease of storage, having reduced post-combustion pollutant emissions, and being renewable.^7−10^ Moreover, some biodiesels can show similar spray patterns and combustion properties to those of conventional diesel fuels^11,12^ and can be directly utilized in diesel engines with little or no modifications.^13,14^ A further advantage of biodiesel is that each fuel has its characteristics depending on the feedstock.^15^

The fuel performance of biodiesels can be investigated in conventional (or partially modified) diesel engines concerning their power production performance and emission characteristics. Numerous studies analyzed biodiesels in terms of their brake thermal efficiency and brake specific fuel consumption. Previous research studies^16−20^ showed that fuel consumption increases with the utilization of neat biodiesel or diesel/biodiesel blends in diesel engines. This is because of comparatively lower calorific values, higher densities, and viscosities of certain biodiesels than conventional diesel. On the other hand, emissions from diesel engines tend to decrease with the utilization of biodiesel–diesel blends. It is a common conclusion that biodiesels emit lesser CO, HC, and PM than conventional diesel fuels when utilized in diesel engines.^18,19,21−23^ This is because inbuilt oxygen content of biodiesels enhances the combustion process, resulting in decreased CO, HC, and PM emissions. However, the situation is not the same for NO_*x*_ emissions. Many studies^23−27^ showed that NO_*x*_ emissions increase when biodiesels are utilized instead of neat diesel fuels. This increase can also be explained by inbuilt oxygen present in biodiesels, where better combustion resulting from inbuilt oxygen content leads to higher in-cylinder temperatures causing nitrogen to react with oxygen. The increase in NO_*x*_ emissions is not a desirable consequence. However, overall, the decrease in other pollutant emissions such as CO, HC, and PM is a good sign for the environment. In addition, the overall biodiesel production cycle can show carbon-neutrality since carbon emitted during biodiesel combustion can be captured during the growth of cultivated biomass used to produce biodiesel.^18^ Therefore, biodiesels can be helpful to mitigate the impacts of climate change.

Since biodiesels are non-toxic and environmentally friendly fuels, as previously explained, research on biodiesels is still ongoing in many aspects. One of the research topics is spray investigation. Spray characteristics of diesel-like fuels result from two-phase flow happening during the injection process. It is essential to clearly understand the fuel–air mixing process to improve the performance and reduce the emission levels of diesel engines.^28^ Biodiesels are different from conventional diesel fuels in terms of their physical properties, such as density, viscosity, and surface tension.^29,30^ These properties influence fuel atomization and spray pattern and thus the output performance, fuel consumption, and pollutant emission levels.^31,32^ Knowing the spray characteristics of the biodiesel to be used can provide better performance and emission characteristics with minor changes in the operating parameters of the diesel engine. Different researchers have utilized several test rigs so far to obtain the spray characteristics of fuels. Some examples of these test rigs are optical research engine (ORE), rapid compression machine (RCM), constant pressure flow rigs (CPFR), and constant volume spray chamber (CVSC). More detailed information about these methods can be found elsewhere.^33,34^ Among these test rigs, constant volume spray chambers have the advantage of possessing a wide range of tested gas temperatures and pressures,^33^ and therefore, it is the preferred technique used in this study.

Numerous research works concerning the investigation of spray characteristics of biodiesels can be found in the literature. Table 1 summarizes the available studies in the literature. The spray characteristics of various biodiesels are compared based on the type of feedstock they are produced from and with the fossil diesel fuel by using CVSC. In addition, the table compares the physical properties of biodiesels, such as density, viscosity, and surface tension, with those of diesel. These studies mainly focused on spray penetration length and spray cone angle. According to the majority of the researchers,^32,35−39^ with the utilization of biodiesels instead of conventional diesel fuel, the spray penetration length increases, and spray cone angle decreases. For example, Yu et al.^35^ experimentally investigated the spray characteristics of biodiesel derived from waste cooking oil in a CVSC under injection pressures of 50, 70, and 90 MPa and chamber pressures of 1, 2, and 3 MPa. They found smaller spray cone angles for the biodiesel under all experimental conditions. In addition, they found that the average spray cone angle for the biodiesel was 20.8% smaller than that of the diesel fuel at 1 ms after the start of injection, under the chamber pressure of 3 MPa and the injection pressure of 90 MPa. Furthermore, they found that biodiesel spray penetration was always longer than diesel spray penetration during the entire injection duration under all experimental conditions. Longer spray penetrations and narrower spray angles for biodiesels can be explained by higher density, leading to increased spray momentums, and viscosity and surface tension values of biodiesels than that of the conventional diesel resulting in poor atomization.^32,35−39^ On the other hand, several researchers^40−42^ found similar spray characteristics in terms of spray penetration length and spray cone angle for both biodiesels and conventional diesel fuels. For instance, Patel et al.^40^ examined the spray characteristics of Jatropha biodiesel in a CVSC. They found no distinct differences in spray penetration length and spray cone angle values of biodiesel and diesel, particularly at higher ambient pressures (10 and 20 bar).

**Table 1:** Literature Studies Investigating the Spray Characteristics of Biodiesels in Comparison to those of Fossil Diesel Fuel Using CVSCs^a^

			physical properties			spray characteristics	
reference	biodiesel feedstock	percentage of biodiesel in the tested biodiesel/diesel fuel mixture	ρ	ν	σ	SPL	SCA
(32)	rapeseed	10, 20, 30, 40, 50%	↑	↑	—	→	→
		100%	↑	↑	—	↑	↓
(35)	WCO	100%	↑	↑	↑	↑	↓
(36)	Karanja	40%	↑	↑	—	→	→
		60, 100%	↑	↑	—	↑	↓
(37)	WCO	20, 100%	↑	↑	—	↑	↓
(38)	1-Jatropha	100%	↑	↑	↑	↑	↓
	2-palm oil	100%	↑	↑	↑	↑	↓
(39)	1-palm oil	100%	↑	↑	↑	↑	↓
	2-WCO	100%	↑	↑	↑	↑	↓
(40)	Jatropha	20, 100%	↑	↑	↑	→	→
(41)	Karanja	20, 40%	↑	↑	—	→	→
(42)	WCO	100%	↑	↑	—	→	→

a Abbreviations and symbols: ρ: density, ν: kinematic viscosity, σ: surface tension, SPL: spray penetration length, SCA: spray cone angle, WCO: waste cooking oil, 100%: pure biodiesel, ↑: increase relative to diesel, ↓: decrease relative to diesel, →: similarity with diesel, —: no data available.

This study aimed at investigating the spray characteristics of various biodiesels produced from canola, corn, cottonseed, and sunflower oils in terms of spray penetration length and spray cone angle. These feedstocks were selected since they are widely available in Turkey and can be easily procured. In addition, they are among the prevalent resources for biodiesel production.^43^ Corn and canola were the second and the third largest feedstock inputs for biodiesel production in the US in 2019, respectively. Around 798 million liters of corn oil biodiesel and 560 million liters of canola biodiesel were produced.^44^ Sunflower oil is one of the biodiesel feedstocks used in Europe, and approximately 245 thousand metric tons of sunflower oil were utilized to produce biodiesel in 2019.^45^ Cottonseed usage is less for biodiesel production in Europe than other feedstocks;^45^ however, it is a stable source for biodiesel production owing to the content of saturated fatty acid (ca. 29.6%).^43^ Therefore, these resources have been worth investigating. Although several studies concerning the combustion, performance, and emission analysis of biodiesels obtained from canola, corn, cottonseed, or sunflower oils can be found in the literature,^46−52^ studies regarding the spray investigation of biodiesels derived from these resources are very few considering the importance of spray investigation as explained above. The available studies are only limited to the works conducted by Lee et al.^51^ and Kim et al.^52^ In both research studies concerned, the spray characteristics of biodiesel obtained from only canola oil among the above-mentioned resources were investigated. In addition, these research papers included only the effects of injection pressure on spray characteristics. Unlike these studies, the present article included the impact of ambient pressure, which is one of the most critical parameters in spray research, in addition to the effects of injection pressure. Moreover, there was not yet a published study focusing on spray characteristics of biodiesel fuels derived from corn, sunflower, or cottonseed oils while this study was being prepared. Hence, this paper intends to fill the stated gaps in the literature by examining the spray characteristics of canola oil, corn oil, cottonseed oil, and sunflower oil methyl esters in a CVSC under variable ambient and injection pressures.

## 2 Methods

### 2.1 Materials and Biodiesel Production

In this work, four different types of methyl ester biodiesels were used. Biodiesels were produced through the transesterification process, in which the organic group of alcohol takes the place of the organic group of an ester.^53^ The reaction of ester occurs with an alcohol such as methanol, ethanol, etc., in the presence of a catalyst such as KOH, NaOH, etc. General information about the transesterification of vegetable oils can be found in the study by Schuchardt et al.^54^

Three of the methyl ester biodiesels used in this work were produced by using methanol as the alcohol and KOH as the catalyst from canola (Aysan-Soyyigit Group), corn, and sunflower oils (Orkide-Kucukbay Oil and Detergent Inc.) at the Renewable Energy and Hydrogen Research Laboratory of Izmir Institute of Technology. All biodiesels were produced by applying the same methodology as explained in the following. One liter of vegetable oil reacted with alcohol in a 6:1 molar ratio of alcohol to lipid in the presence of 1 wt % of catalyst to lipid. All reactions were carried out using a magnetic stirrer (WiseStir MSH-20D). The transesterification process began by adding the catalyst (KOH) into the alcohol (methanol) under room conditions. Then, the solution started to be stirred at 1100 rpm, and its temperature increased to 50 °C. After the temperature of the alcohol–catalyst solution reached 50 °C, this solution was stirred at 1100 rpm for a further 10 min. At the same time, vegetable oil was heated up to 50 °C. In the next step, vegetable oil at 50 °C was added to the alcohol–catalyst solution at 50 °C. The reaction took place for 240 min at a stirring speed of 1100 rpm. After the reaction ended, the mixture sat for 4 h until the precipitation of glycerol formed as a byproduct. Then, biodiesel was separated from the glycerol. After obtaining the biodiesel, the washing step was applied to improve the biodiesel quality. A total of 5 vol % acetic acid solution in water was prepared, and the pH of the acetic acid solution was measured. Then, 1/3 vol % acetic acid solution was added to the biodiesel so that the ratio of the acetic acid solution and biodiesel to be washed was 1/3 in volume. Next, this mixture was stirred at 500 rpm for 60 min. After 60 min, water was separated from biodiesel. These steps were repeated until the pH of the acetic acid solution in water removed from the biodiesel washed was the same as the bulk acetic acid solution. After reaching the required pH value, the washing process was finished. In the next step, the vacuum evaporation process was applied to the biodiesel at 75 °C for 24 h to remove the remaining water molecules. Finally, biodiesel fuel was prepared for use.

The fourth type, cottonseed oil-based biodiesel, was purchased from DB Tarımsal Enerji Inc. as commercial biodiesel. A commercial diesel fuel (Shell V-Power Diesel with a product code of 002D2609), procured from Shell & Turcas Petrol, was used as the reference fuel. More information about the diesel fuel can be found in its datasheet.^55^

### 2.2 Fuel Properties

**Figure 1:**
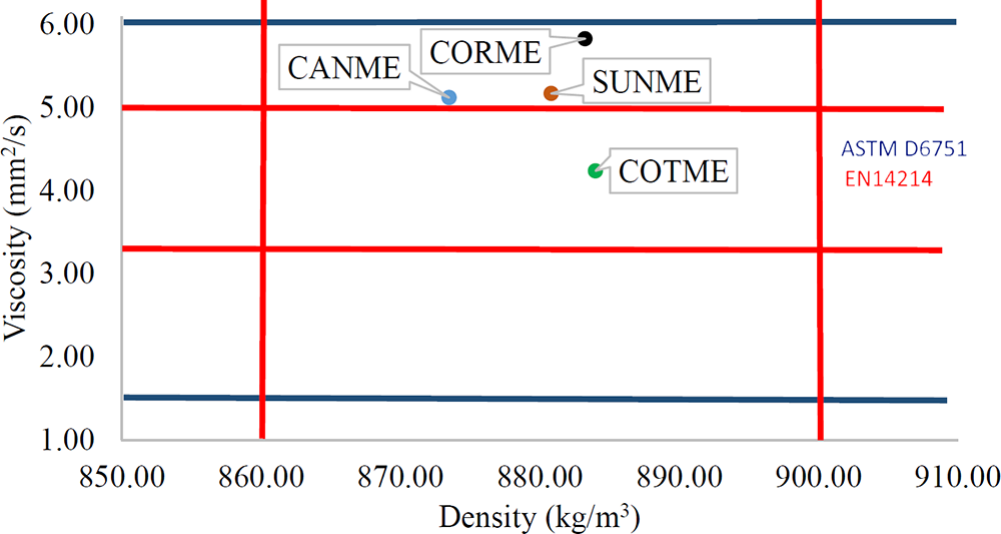
Comparison of the physical properties of the biodiesels with the EU and the US standards.

The methyl ester biodiesels are referred to as CANME, CORME, COTME, and SUNME based on their feedstocks, which were canola, corn, cottonseed, and sunflower oils, respectively. Table 2 shows the physical properties of the tested fuels. Figure 1 shows a comparison of the physical properties of biodiesels with respect to European and American standards. ASTM D6751 standard applies for biodiesel fuels in the USA, and the viscosity value of biodiesel must be between 1.9 and 6 mm^2^/s according to this standard.^56^ EN 14214 applies to biodiesel in the EU, and biodiesels must meet these standards by having density values between 860 and 900 kg/m^3^ and viscosity values between 3.5 and 5 mm^2^/s.^57,58^

**Table 2:** Properties of the Fuels Tested in this Study

test fuel	viscosity (mm^2^/s) @ 40 °C	density (kg/m^3^) @ 15 °C	contact angle (°) with glass @ 25 °C
diesel	3.07	829.55	14.71
CANME	5.12	873.50	20.16
CORME	5.83	883.26	20.46
COTME	4.24	884.01	20.23
SUNME	5.17	880.03	20.34

The densities of fuels were measured using a calibrated pycnometer at 15 °C. The total volume of the pycnometer is 25.066 mL, and it is calibrated with 0.001 mL sensitivity with its lid. A thermometer whose sensitivity was 0.1 °C and an accuracy of ±1 °C was used to measure the temperature of the fuels during density measurements.

The viscosities of the fuels were measured using an AR 2000ex rheometer, which TA Instruments developed. The rheometer was used in controlled rate mode. The angular velocity of the rotating plate in the rheometer was 10.5 rad/s, and the fuel temperature was brought to 40 °C. Moreover, the rheometer received 1 data in 5 s and a total of 60 data for each experiment.

The contact angle values of all fuels were measured to predict the surface tension effects. They were measured using a Theta optical tensiometer, developed by Attension, at room temperature. The measurement accuracy of the device was ±0.1°. In addition, glass was used as the solid material to obtain the contact angles.

### 2.3 Experimental Setup

**Figure 2:**
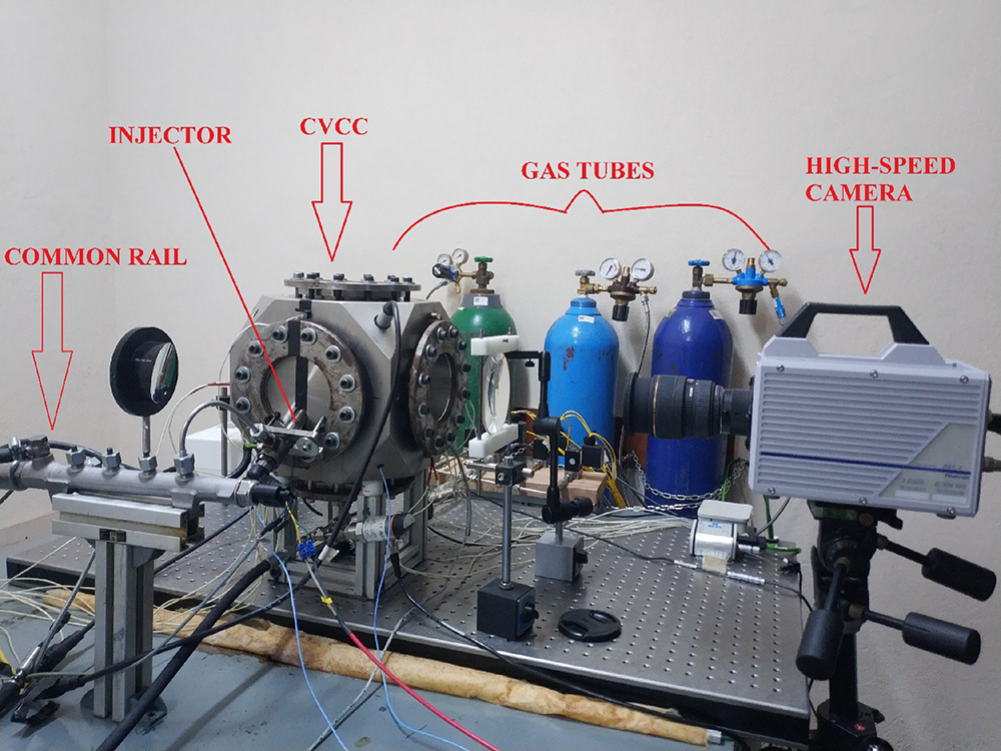
The spray test rig.

All experiments were performed in a constant volume spray chamber (CVSC) seen in Figure 2. In this experimental setup, two different spray measurements can be made, reactive and non-reactive. A reactive environment can be obtained by adding a flammable gas and air into the chamber, and a non-reactive environment can be formed by filling the chamber with only air. In this study, a non-reactive environment was used because it was aimed at observing how the physical properties of the biodiesel fuels affect the spray propagation under various experimental conditions.

**Figure 3:**
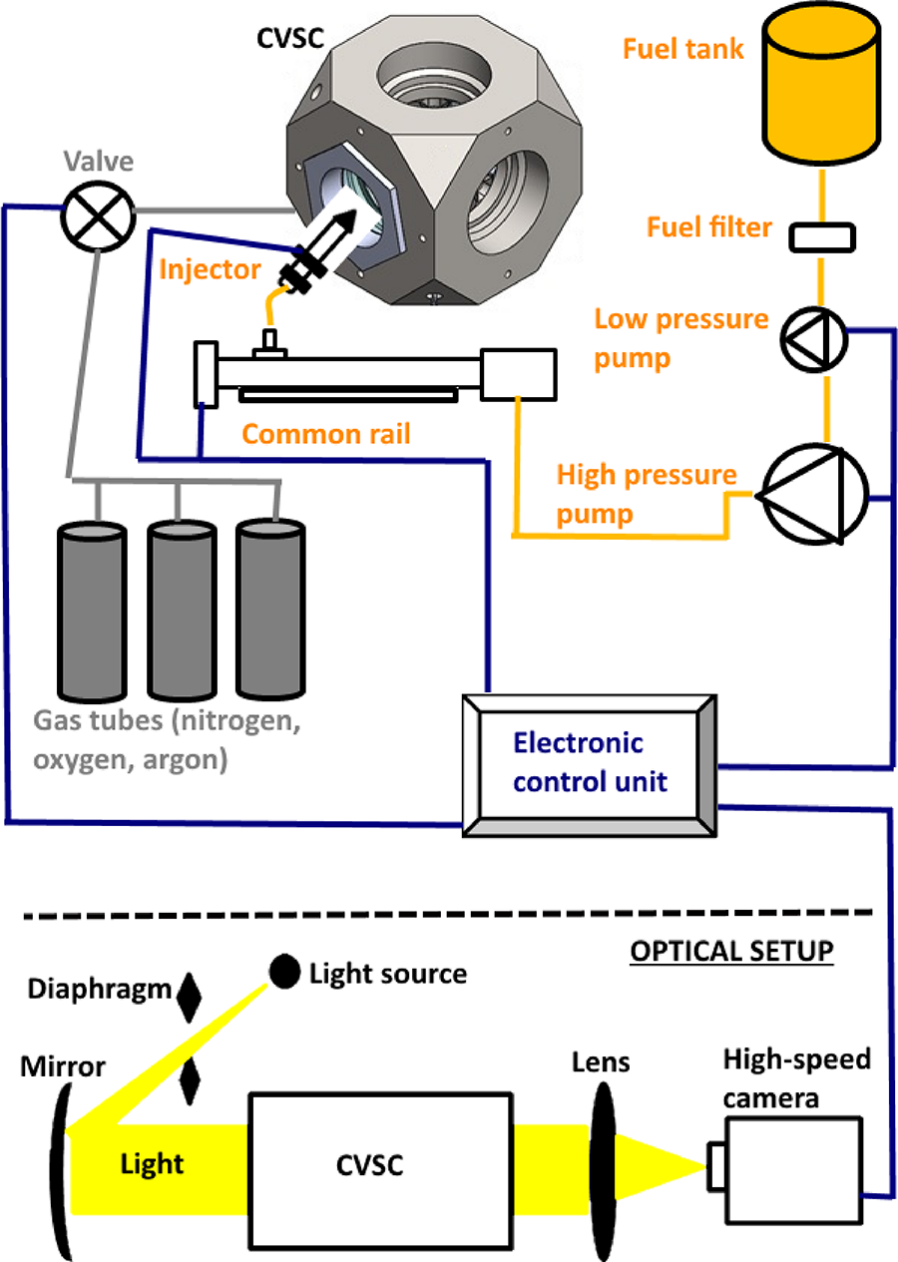
Schematic sketch regarding the subsystems of the constant volume spray chamber.

For injecting the fuels into the CVSC, a fuel injection system was assembled in the test rig, as shown in Figure 3. The fuel injection system consisted of a fuel tank, low-pressure pump, fuel filter, high-pressure fuel pump, common rail, and an injector. The fuel pump was operated by an electric motor whose power was 2.2 kW. A Siemens common rail diesel injector with a model number A2C59517051 (and nozzle part number: M0019P140) was utilized in the experiments. The injector originally had eight injection holes on its tip. However, seven holes were closed by laser welding, and one hole was left open so that a single spray formation could be observed.

CVSC had optical quartz windows having diameters of 120 mm for spray visualization via various optical techniques, allowing the observation of the entire spray process. The maximum length between the injector nozzle hole and the chamber wall was 102 mm on the spray axis. In this work, a shadowgraph system was installed that could directly detect the liquid phase of the spray, as demonstrated in Figure 3. A high-speed camera (Photron Fastcam SA1.1), whose properties are listed in Table 3, was utilized to visualize the spray process. The camera was equipped with a Sigma lens (Sigma 24–70 mm f/1:2.8). The spray videos were recorded at 20,000 fps and a shutter speed of 1/62,000 with a resolution of 512 × 512 pixels. In addition, a Dolan Jenner Fiber-Lite MI-150 Illuminator was used as the light source.

**Table 3:** Specifications of the High-Speed Camera

Photron Fastcam SA1.1	
image sensor	CMOS image sensor
sensor resolution	1024 × 1024 pixels
frame rate	max. 5400 fps for full resolution
	max. 675,000 fps for reduced resolutions
recording color depth	monochrome (12 bit)
	color (RGB, each 12 bit)
shutter method	electronic shutter
trigger method	start, center, end, manual, random, random reset, random center, random manual, two-stage
trigger input signal	TTL, contact

Control of the equipment in the test rig was performed via a control system consisting of a computer, a National Instruments (NI) USB 6343 data acquisition (DAQ) card, and a NI USB 6353 DAQ card. Processing of the data incoming to the control system and giving the necessary commands was performed by the programs prepared in the Labview software.

Table 4 shows the pressure and temperature measurement devices utilized in the constant volume spray chamber with accuracy values. The total uncertainty of the experiments was calculated with the method of propagation of errors defined by Holman, taking the square root of the sum of the squares of all accuracy values.^59^ The total uncertainty was ±0.84%.

**Table 4:** Specifications of the Measurement Devices Used in the Study

equipment	parameter	range (units)	accuracy
Kistler 4075A50V200S	pressure	0–50 (bar)	%0.1
Kistler Piezoresistive Amplifier Type 4624A	voltage output	0–10 (V)	%0.05
	error of the electronics		%0.75
MAX6675 K-type thermocouple	temperature	–20–80 (°C)	%0.25
Emko ESM-4420 temperature control device	temperature control	0–50 (°C)	%0.25

### 2.4 Test Conditions

During the experiments, three different injection pressures were utilized, which were 600, 800, and 1000 bar. These injection pressures were selected because these values were the values the experimental setup already installed could achieve. The fuel pump was not stable at higher injection pressures. In addition, fuels impinge on the wall of the spray chamber at pressures like 1200–1800 bar, and thus, it is difficult to observe the differences in spray characteristics of the test fuels. In addition, the ambient pressure was adjusted from 0 to 15 bar by increasing the pressure by 5 bar. 0 bar (absolute) and 5 bar chamber pressures were relatively lower. These pressure values were used to observe the mixing effect of the air on different fuels since the kinematic viscosity of air changes with pressure. Test conditions are presented in Table 5. Different studies used the selected (or close) ambient and injection pressures.^35,38,60,61^

**Table 5:** Test Conditions Utilized in the Study

condition	property
number of the nozzle holes	1
nozzle hole diameter (μm)	200
injection duration (ms)	1
injection pressure (bar)	600, 800, 1000
absolute chamber pressure (bar)	0, 5, 10, 15
chamber temperature (°C)	25
repetition of the experiments	5

### 2.5 Image Processing

Spray images obtained from experiments were processed to accurately measure spray penetration length, spray cone angle, and spray area. Spray penetration length is the maximum length between the injector nozzle and the farthest point on the spray axis. Spray cone angle is defined as the angle between the nozzle and the two farthest apart points on the outer spray boundary, and two outer points can be located at 60% of the spray penetration length.^35^ The spray area is the region enclosed by the spray boundaries.

**Figure 4:**
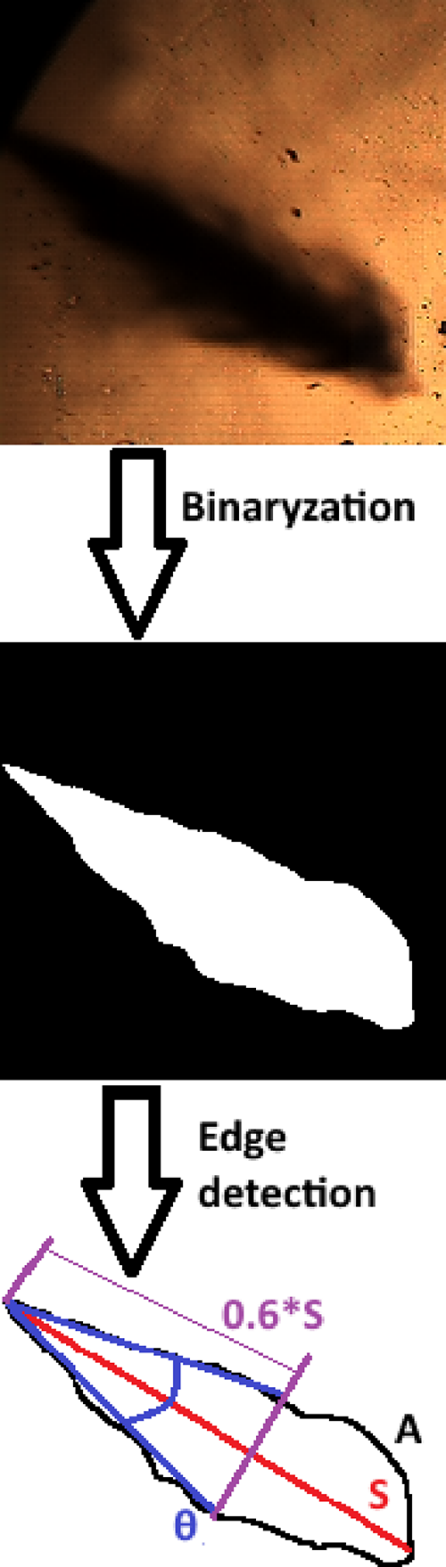
Method of image processing.

Image processing was performed by using ImageJ.^62^Figure 4 shows the method of image processing, where *S* is the spray penetration length, θ is the spray cone angle, and *A* is the spray area. Firs, background subtraction was done to raw spray images to obtain isolated spray images. In the second step, thresholding was performed on the isolated spray images to convert these images into a binary scale. Then, edge detection was applied to binary images. Finally, required spray parameters such as spray penetration length and spray cone angle were measured from the processed spray images.

## 3 Experimental Results and Discussions

**Figure 5:**
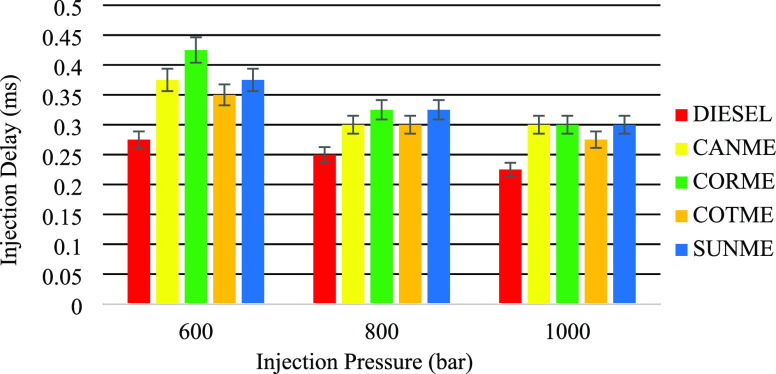
Injection delay.

**Figure 6:**
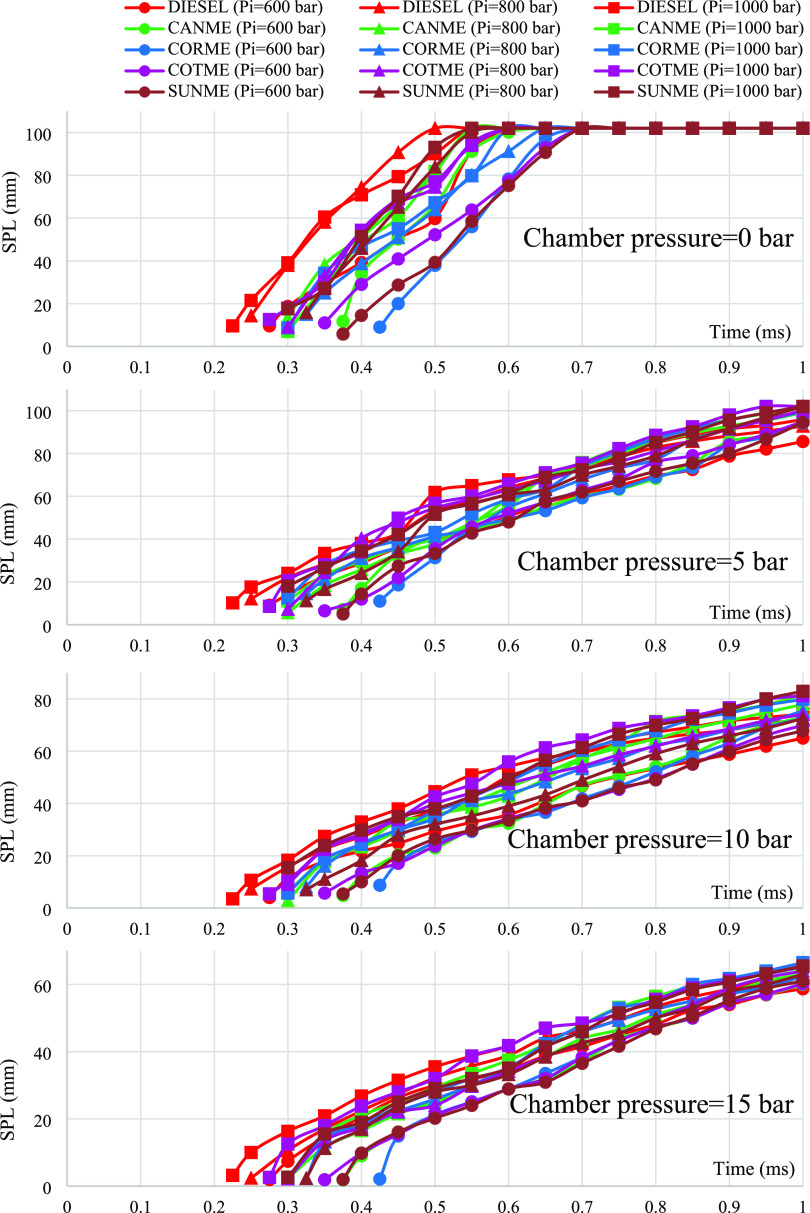
Variations in spray penetration lengths for biodiesels in comparison with diesel.

**Figure 7:**
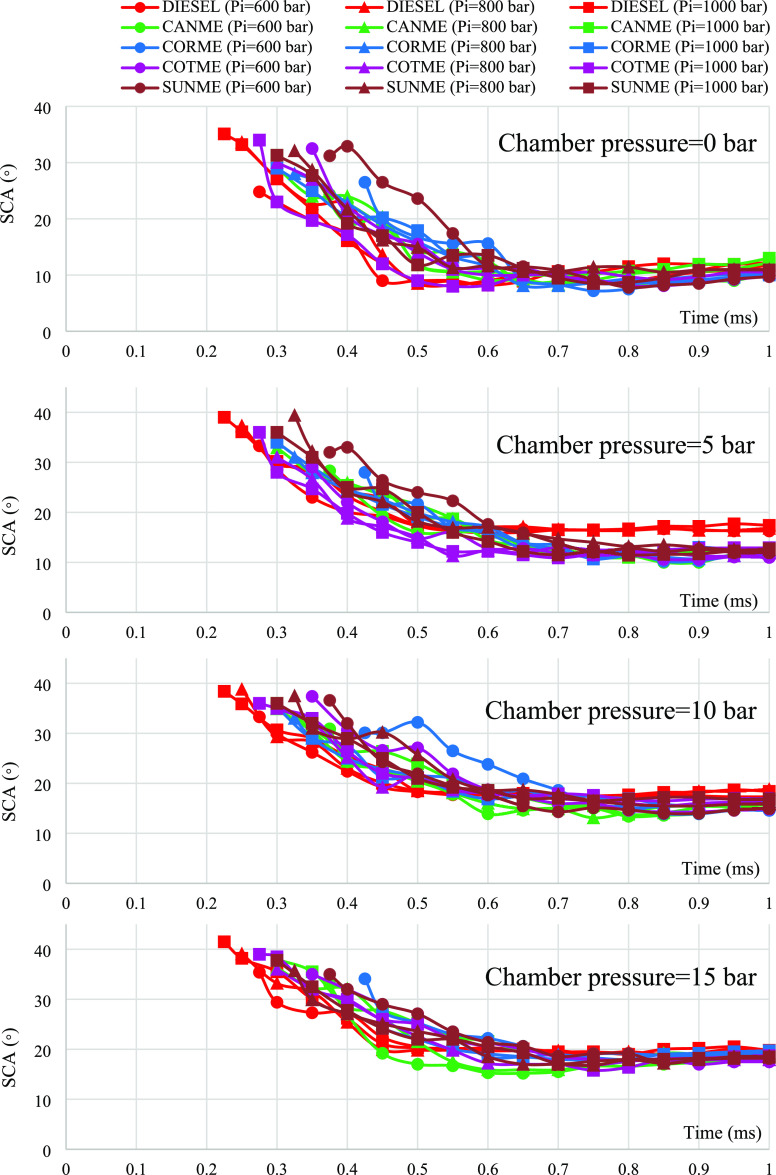
Variations in spray cone angles for biodiesels in comparison with diesel.

**Figure 8:**
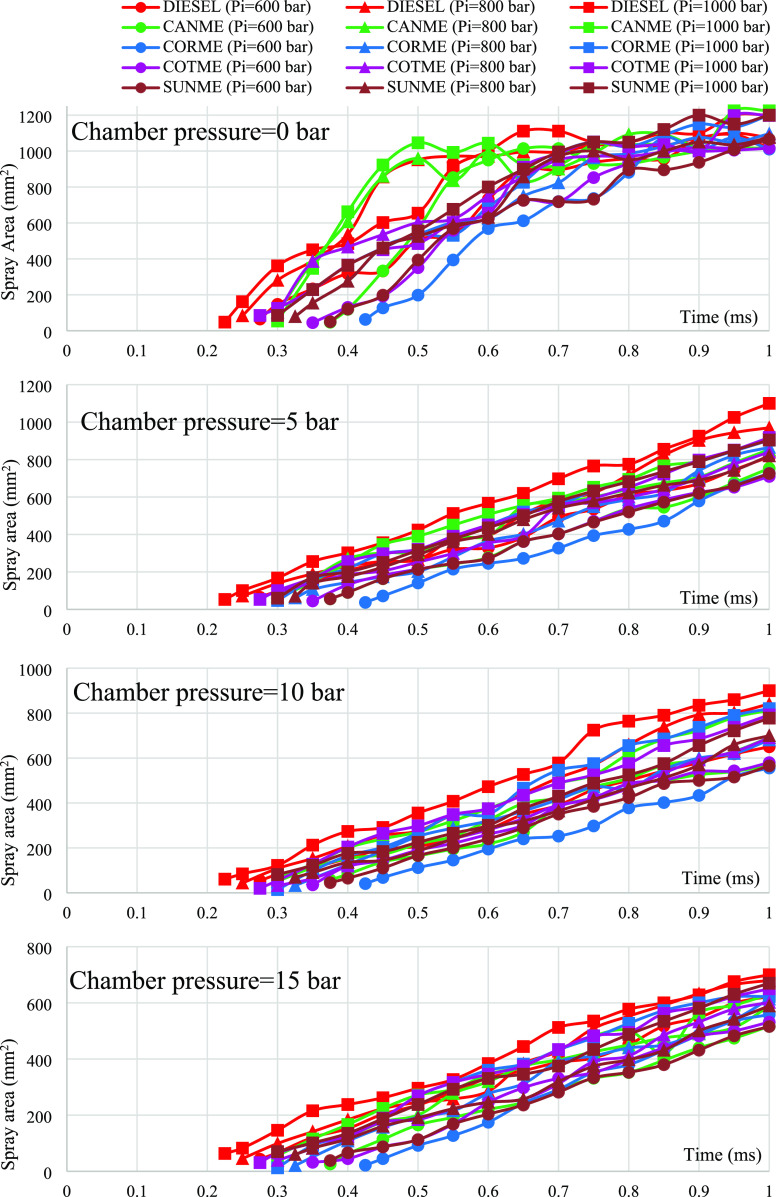
Variations in spray areas of biodiesels in comparison to those of fossil diesel.

Figures 5–8 show the effects of fuel properties on spray characteristics under specified conditions in a CVSC. Each experiment corresponding to each test condition was repeated at least five times. The results presented in the figures are the mean values of these five experiments. The results of spray analysis demonstrated that the deviation of the experimental values from the mean values for spray characteristics was within 5%, and this value will be referred to as repeatability in the following. Repeatability is shown in the graphs instead of uncertainty because the repeatability value is more significant than the uncertainty value.

### 3.1 Injection Delay

Injection delay is the time between the trigger signal energizing the injector and the start of the injection process. Approximate injection delays can be determined by detecting the first spray image. Since the energizing time of the injector is known to be at 0 ms, the frame at which the first spray image is obtained gives the approximate injection delay time.

Figure 5 shows the injection delays of the fuels corresponding to three injection pressures. Injection delay was affected by injection pressure, and it decreased as the injection pressure increased. For example, injection delays of diesel fuel were 0.275, 0.25, and 0.225 ms under injection pressures of 600, 800, and 1000 bar, respectively. This could mainly be due to the accelerated injector needle lift movement by the raised injection pressure.^39^ Moreover, biodiesel fuels performed longer injection delays than fossil diesel. For instance, the injection delay of CORME was around 0.075 ms longer than that of diesel under the injection pressure of 1000 bar. Higher viscosities of biodiesels than diesel can cause higher frictions around the injector needle, and this can result in slower needle lift movement causing longer injection delays.^63^ This result is consistent with the several literature studies.^30,39,63−65^

### 3.2 Spray Penetration Length

Figure 6 shows the effects of fuel properties on spray penetration length under the variable chamber and injection pressures. As shown in the figure, spray penetration curves of biodiesels are similar to those of conventional diesel fuel. As a general inference, increasing the injection pressure raised the spray penetration lengths of all fuels while raising the chamber pressure decreased the spray penetration lengths.

At zero chamber pressure, considering the uncertainty and repeatability values, no significant distinctions between biodiesels and conventional diesel fuels were observed. This was the case for all injection pressures (i.e., 600, 800, and 1000 bar). This could be explained by the fact that sprays of all fuels penetrated very fast and impinged on the chamber wall. There might have been no time for the difference to occur. In addition, there was nothing inside the chamber for the fuels to mix with, and thus, no difference occurred.

At chamber pressure of 5 bar, considerable differences were found between the penetration lengths of biodiesels and diesel for all injection pressures. At the beginning of the injection process, diesel fuel performed longer penetration lengths than biodiesels due to having shorter injection delays. This situation was valid for all injection pressures. However, biodiesels performed longer penetrations than diesel toward the end of the injection process. For example, when comparing COTME and diesel in terms of SPL under injection pressure of 600 bar, it was observed that the measured penetration lengths that COTME and diesel achieved were 35.5 and 40.3 mm at 0.5 ms of the injection process. However, COTME reached 94.9 mm of SPL at the end of the injection, while the diesel reached 85.6 mm of SPL. In addition, all biodiesel fuels impinged on the chamber wall at the end of injection at injection pressures of 800 and 1000 bar. On the other hand, diesel fuel did not hit the wall in either injection pressure condition.

At chamber pressure of 10 bar, all fuels, including biodiesels, did not impinge on the chamber wall under all injection pressure conditions. In addition, the difference between penetration lengths of diesel and biodiesels reduced, although biodiesels still had slightly bigger values. A previous example compared diesel and COTME at 5 bar chamber pressure. When the ambient pressure increased from 5 to 10 bar, the distinction between the maximum penetration lengths of these fuels decreased to 4.9 from 9.3 mm. The results showing longer penetrations for biodiesels are similar to those found by Tinprabath et al.^32^ and Yu et al.^35^ Both studies reported that biodiesel fuels had longer spray penetration lengths than diesel under different experimental conditions. Higher penetration lengths of biodiesels could be explained by having higher density, viscosity, and contact angle values than diesel fuel. Having higher density could lead to larger momentum resulting in deeper penetrations.^38^ Despite their longer injection delays, higher momentums of biodiesels resulted in increased spray penetrations. Higher viscosity could adversely affect fuel spray atomization, resulting in increased liquid penetrations.^37^ Also, surface tension effects were more prominent for biodiesel fuels, considering the higher contact angle values, resulting in poor atomization and increased liquid lengths.^35^ In addition, biodiesels generally have higher boiling points making them less volatile than diesel, resulting in longer SPLs.^28,66,67^

When chamber pressure further increased to 15 bar, the differences between the SPLs of biodiesel and diesel fuels decreased more and became insignificant. For example, the difference between spray penetration lengths of COTME and diesel fell to 1.2 mm under an injection pressure of 600 bar at the end of the injection process. When considering repeatability and uncertainty ratios, this difference was not significant. This result is different from several other research studies,^32,35,37,38,40^ which reported increased spray penetration lengths for biodiesel fuels under ambient pressures higher than 10 bar.

The decreasing trend in the difference between SPLs of diesel and biodiesels and becoming similar could be explained by the kinematic viscosity of air. At zero chamber pressure, there was no air to cause neither shear drag nor turbulent mixing. There might be flashed boiled fuel at zero pressure, but this might move with the same speed as the fuel itself, resulting in no drag. This might be why there was no difference in spray penetration lengths of biodiesel and diesel at 0 bar. The situation was different when there was air inside the chamber. Kinematic viscosity of air monotonically gets relatively smaller since air density was getting larger due to pressure rise in the chamber. The air’s smaller kinematic viscosity made the airflow around the fuel jet more prone to turbulence, and turbulent air increased mixing. Thus, turbulent air around the fuel jet might be causing a larger spread of diesel at 5 bar than at 0 bar. The same argument was valid for biodiesels because they also spread more in the radial axis at ambient pressure of 5 bar than at zero chamber pressure. However, there were significant differences between biodiesel and diesel sprays at 5 bar. Biodiesel fuels had larger surface tension values than diesel. Thus, biodiesels did not disintegrate as quickly as diesel. Eventually, spray penetration lengths of biodiesels were longer than that of diesel. Furthermore, as the chamber pressure increased, the kinematic viscosity of air got smaller. Air turbulence might be becoming high enough to mix biodiesels as well as diesel effectively. Therefore, differences between the spray penetration lengths of diesel and biodiesels were reduced.

### 3.3 Spray Cone Angle

Figure 7 demonstrates the effects of fuel properties on spray cone angle under the various chamber and injection pressures. As a general inference, spray cone angles increased when ambient pressure rose. In contrast, a slight increase in spray cone angle occurred with an increase in injection pressure, which could be assumed to have remained almost constant considering the uncertainty and repeatability. When comparing biodiesels with diesel fuel, different results were obtained depending on the experimental conditions. Two different trends were observed in spray cone angle curves. SCA values were high at the first stages of the injection. Then, they decreased to a value at which SCA was almost constant. Due to the injection delay, biodiesels reached this steady value later than diesel.

At zero ambient pressure, no essential differences between the spray angles of biodiesels and diesel fuel were observed for both injection pressures when considering the uncertainty and repeatability ratios. All fuel sprays impinged on the chamber wall approximately in 0.4 ms when they entered the spray chamber, that is, in the early injection stages. Namely, there was insufficient time for sprays to develop before impinging the wall. In addition, there was no air inside the chamber to mix the fuels at zero ambient pressure. Therefore, differences between SCAs of biodiesels and diesel could not have occurred.

However, significant distinctions were obtained when the chamber pressure increased to 5 bar. For example, SCA of diesel at the injection pressure of 800 bar was 16.8° at the end of the injection, while SCA of SUNME was found as 12.6° under the same conditions. This value corresponds to a 25% lower SCA for SUNME. When the injection pressure increased to 1000 bar at the same ambient pressure, the difference between the SCAs of SUNME and diesel yielded almost the same result as the test at the injection pressure of 600 bar with a 25.8% distinction.

At the chamber pressure of 10 bar, the difference between the spray angles of diesel fuel and biodiesels slightly decreased compared to the values at the chamber pressure of 5 bar, and biodiesels still had smaller values than the reference diesel. For instance, spray cone angles of diesel and SUNME at the injection pressure of 800 bar were found as 18.7 and 16.1°, respectively at the end of the injection. Namely, the difference decreased to 2.6 from 4.2° when the chamber pressure was raised to 10 from 5 bar. The results showing lower SCA values for biodiesels are consistent with those found by Xie et al.^60^ That study reported decreased spray cone angle values for biodiesel fuels compared to fossil diesel under different experimental conditions such as chamber pressures between 1 and 9 bar and injection pressures between 600 and 1000 bar. Narrower spray cone angles for biodiesel fuels might be due to their higher viscosity and contact angle values (higher surface tension effects), resulting in poor atomization.^38,60^

Moreover, the differences in SCAs of biodiesels and diesel were further reduced as the ambient pressure increased to 15 bar and could be neglected considering the uncertainty and repeatability values. For instance, the difference between diesel and SUNME decreased to 0.8° at the end of the injection process (800 bar). As another example, the commercial biodiesel (COTME) and diesel yielded similar results in terms of SCA with 19.1 and 19.8°, respectively. This result showing the similarity between biodiesels and fossil diesel is different from literature studies,^32,35,37,38,40^ which presented lower SCA values for biodiesels than diesel under similar test conditions to the present study. The effect of chamber pressure on spray angle can be explained with the same reason given for spray penetration lengths. As the air pressure inside the spray chamber increases, the air may similarly mix the two types of fuel.

When biodiesels were compared among themselves in terms of spray penetration length and spray cone angle, minor differences in these properties were observed. However, these slight distinctions had no point when considering the uncertainty and repeatability ratios. Namely, it could be stated that all biodiesels yielded similar SPLs and SCAs with negligible differences. This could be explained by all biodiesels’ comparable density, viscosity, and contact angle values. This result is similar to that of Deng et al.^38^ They investigated the spray characteristics of different biodiesel fuels derived from Jatropha and palm oils with comparable physical properties such as density, viscosity, and surface tension in a constant volume vessel. They found similar SPLs and SCAs for different biodiesel fuels under variable injection pressures (60, 90, 120, and 150 MPa) and ambient pressures (1.1, 2.1, and 3.1 MPa). However, their results are different from the present study when considering the comparison of biodiesels with conventional diesel. They found longer spray penetrations and narrower spray angles for biodiesels under all experimental conditions. The present study showed that SPLs and SCAs of biodiesel fuels were almost identical to those of diesel at relatively higher ambient pressures, e.g., 15 bar.

### 3.4 Spray Area

Figure 8 demonstrates the influences of fuel properties on spray areas under different ambient and injection pressures. As presented in the figure, spray area curves of biodiesels are similar to those of petroleum diesel fuel. As a general inference, the spray area increased as the injection pressure rose and chamber pressure diminished.

At zero chamber pressure, spray area curves quickly increased to a maximum value. Then, spray area values continued thereabout steadily until the end of the injection process around the maximum value, although small fluctuations occurred. In addition, differences between the spray areas of biodiesels and diesel were not significant when considering the uncertainty and repeatability, especially in the maximum spray areas. No spray development stage was observed as the sprays hit the chamber wall very quickly without the presence of air.

At the chamber pressure of 5 bar, significant differences existed between the biodiesels and fossil diesel in terms of spray area as in SPL and SCA. For instance, the maximum spray area covered by the diesel fuel at the injection pressure of 1000 bar is around 1100 mm^2^, while the spray area of CANME was at most 905.3 mm^2^ at the same condition, which corresponds to a 17.7% difference.

When the chamber pressure increased to 10 bar, the distinction between biodiesels and diesel still existed, but the difference decreased to a lower degree. For example, the distinction between the maximum spray areas covered by CANME and diesel diminished to 9.7% at the injection pressure of 1000 bar. The result indicating lower spray areas for biodiesels than fossil diesel at the same instant of the injection duration was mainly due to the injection delay. Biodiesels reached similar values to diesel in the later stages of the injection process. In addition, although SPL values of biodiesels were higher than diesel, their SCA values were lower, which led to lower spray area values.^39,61^ Consequently, biodiesels’ larger viscosity and surface tension effects caused lower spray areas than diesel under chamber pressures of 5 and 10 bar, despite their relatively higher SPL values.^65^

Differences in spray areas of biodiesels and diesel further decreased at the ambient pressure of 15 bar. For example, the distinction mentioned above between the largest spray areas of CANME and diesel in the entire injection process decreased to 6.5% at the injection pressure of 1000 bar. However, if the spray area is examined independently of the injection delay, the similarity may be more pronounced. For example, diesel covered an area of 589.3 mm^2^ 0.6 ms after entering the spray chamber under an injection pressure of 1000 bar. Under the same conditions, the spray area of CANME was 563.6 mm^2^ 0.6 ms after entering the spray chamber. Namely, the difference was 4.4%. Considering the uncertainty and repeatability, it can be deduced that spray area values of biodiesels were not much different from those of diesel under a chamber pressure of 15 bar. This may also be explained by the reason given in Section 3.2 for the similarity between the SPLs of biodiesels and diesel. From another point of view, the similarities in the SPL and SCA curves of biodiesels and diesel under 15 bar chamber pressure were confirmed by the similarity obtained in the spray area evaluation.

Despite the slight variations under all chamber and injection pressures, the spray area values of CANME, CORME, COTME, and SUNME were very close to each other. Differences did not exceed 5–6% on average, especially in the latter stages of the injection process. When taking uncertainty and repeatability values into account, these distinctions could be assumed as insignificant.

## 4 Analytical and Empirical Predictions

Experimental results regarding the spray penetration length, spray cone angle, and spray area showed that biodiesels can have deteriorated or similar spray characteristics compared to conventional diesel fuel, depending on the experimental conditions. It was shown that the spray characteristics of biodiesels were worse than those of diesel at the chamber pressures of 5 and 10 bar. However, at the chamber pressure of 15 bar, the spray characteristics of both types of fuels were very similar. To better compare the spray characteristics of biodiesels with those of fossil diesel, several parameters such as spray volume, Sauter mean diameter, and air entrainment were predicted analytically and empirically considering the experimental results under a chamber pressure of 15 bar.

### 4.1 Spray Volume

Spray volume is the atomization volume of a fuel spray, which can provide an insight to predict the air–fuel mixing process. Equation 1 can be utilized to estimate the spray volume:^68^
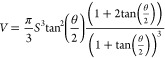
1where *V* is spray volume, *S* is spray penetration length, and θ is spray cone angle.

**Figure 9:**
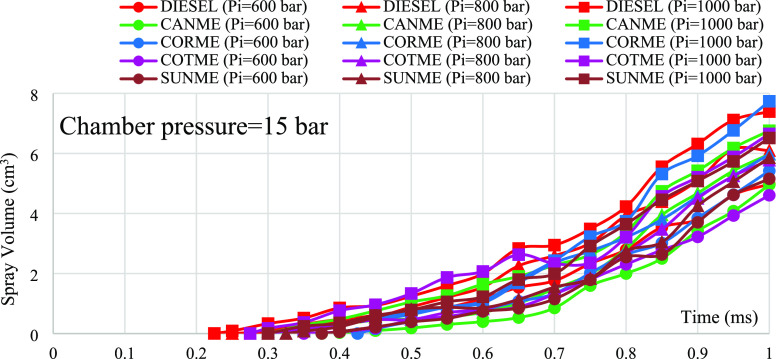
Prediction of spray volume of biodiesels in comparison to that of diesel.

Figure 9 presents the estimation of spray volume values of the test fuels under various injection pressures and a chamber pressure of 15 bar. The figure showed that spray volume increased as the injection pressure rose. This result is inferable since the increased injection pressure increased the spray penetration length and slightly improved the spray cone angle.^60^

Moreover, slight variations occurred between the spray volume values of biodiesels and diesel. For example, the spray volume of diesel and CANME were calculated as 7.4 and 6.9 cm^3^, respectively, at the end of the injection process, which corresponded to a 6.8% difference. On average, differences between diesel and biodiesels were lower than 8% in spray volume.

### 4.2 Sauter mean diameter

Sauter mean diameter (SMD) indicates the average particle size of fuel droplets. It can be predicted by using eqs 2–4 described by Hiroyasu et al.:^69^
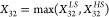
2
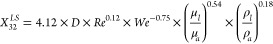
3
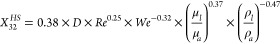
4where *X*_32_ is Sauter mean diameter, *D* is the diameter of injector nozzle, *Re* is the Reynolds number for the fuels, *We* is the Weber number for the fuels, μ_*l*_ is the dynamic viscosity of fuel, μ_*a*_ is the dynamic viscosity of air, ρ_*l*_ is fuel density, and ρ_*a*_ is air density. The Reynolds number can be calculated by using eq 5:
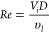
5where *V_i_* is the injection velocity calculated by volumetric flow rate, υ_*l*_ is the kinematic viscosity of the fuel. The Weber number can be calculated using eq 6:
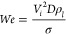
6where σ is the surface tension of the liquid surface. The surface tension values of the fuels were not available; on the contrary, an assumption for surface tension values was made according to the previous research study to predict the possible range for SMD values.^70^ The assumed surface tension values as mean values of several research studies were 27.51 and 31.43 mN/m for diesel and biodiesels, respectively. In addition, the possible error margin according to the highest and lowest surface tension values was calculated and shown in the resulting graph.

**Figure 10:**
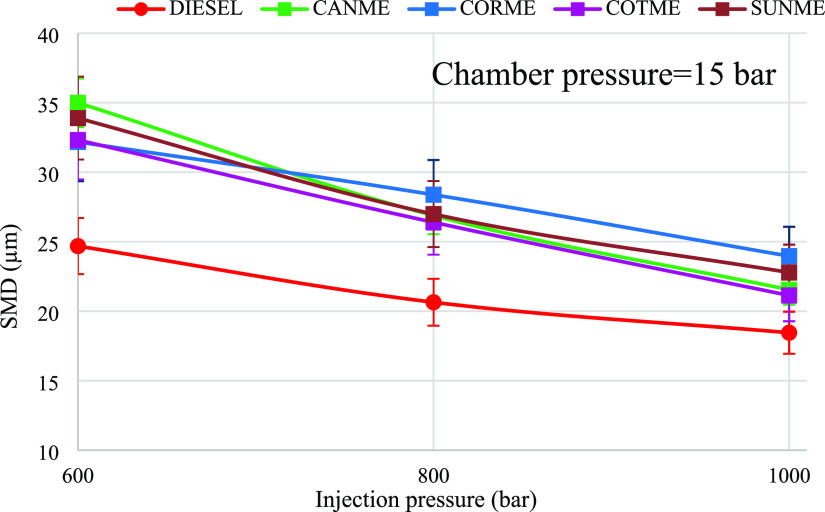
Prediction of Sauter mean diameter for the fuels.

Figure 10 shows the predicted SMD values for the fuels with variable injection pressures under the chamber pressure of 15 bar. The results showed that SMD values decreased with increased injection pressure while keeping the chamber pressure constant. This result is similar to some literature studies.^31,71^ Measured SMD values of biodiesels were higher than those of diesel by 35.1, 31.6, and 21.2% under injection pressures of 600, 800, and 1000 bar, respectively. This was mainly due to biodiesels’ larger viscosity and surface tension values than diesel.^61,72^

### 4.3 Air Entrainment

Air entrainment can be described as the amount of air drawn into the spray structure and somehow indicates the quality of the mixing process of a fuel with air.^37,72^ It can be found using the model described by Rakopoulos et al. (eq 7):^73^
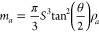
7where *m_a_* is the air entrainment, *S* is the spray penetration length, θ is the spray cone angle, and ρ_*a*_ is air density.

**Figure 11:**
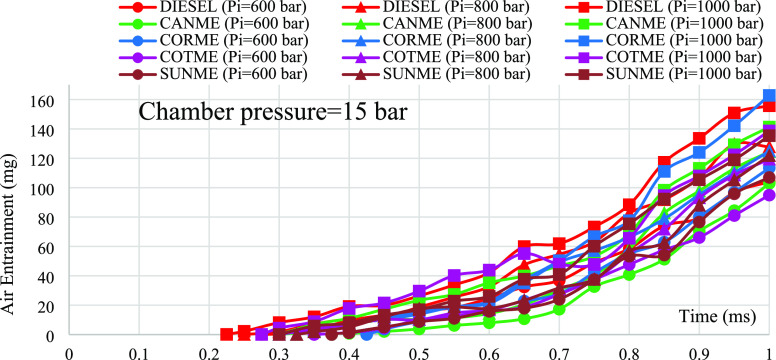
Air entrainment analysis for the fuels.

Figure 11 demonstrates the air entrainment analysis for biodiesels in comparison to those of fossil diesel under a chamber pressure of 15 bar and injection pressures of 600, 800, and 1000 bar. As the injection pressure increased, air entrainment also raised. This is because an increase in injection pressure can improve the atomization.^74^ When comparing biodiesels with diesel in terms of air entrainment, slight differences were obtained. For instance, air entrainment values for CORME and diesel were calculated as 125 and 127.8 mg, respectively, under the injection pressure of 800 bar at the end of the injection process. Namely, CORME had 2.2% lower air entrainment. On average, the distinctions between the diesel and biodiesels were lower than 7.2% under all injection pressure conditions. Slightly reduced air entrainment for biodiesels may be because of their higher viscosity and surface tension values resulting in deteriorated atomization. However, considering repeatability and uncertainty values, it could be deduced that the differences mentioned were not very big.

## 5 Conclusions

In this paper, spray characteristics of biodiesels and conventional diesel fuel were investigated and compared in a constant volume spray chamber under various chamber pressures (0, 5, 10, and 15 bar) and injection pressures (600, 800, and 1000 bar) by using the shadowgraph technique. The main findings of the study can be summarized as follows:

(i) Experimental results showed that biodiesel fuels performed slightly or significantly longer penetrations, narrower spray cone angles, and reduced spray areas compared to reference diesel fuel under chamber pressures of 5 and 10 bar for all injection pressures. This might be due to having higher density, viscosity, and contact angle values resulting in poorer atomization and increased spray momentums. Differences between diesel and biodiesels were found to be around 3–20%, 5–30%, and 5–18% for SPL, SCA, and spray area, respectively, depending on the test conditions.
  However, the situation is different for the chamber pressure of 15 bar. There was no critical difference between biodiesels and diesel in terms of spray penetration length and spray cone angle under both injection pressures, considering the repeatability (±5%) and the uncertainty (±0.84%). On average, the difference did not exceed 3%.
(ii) Furthermore, analytical and empirical predictions were performed to further analyze the spray characteristics of biodiesels at the chamber pressure of 15 bar, which was the pressure at which biodiesels performed similar spray characteristics to diesel. Biodiesels had 35.1, 31.6, and 21.2% higher SMD values than diesel under injection pressures of 600, 800, and 1000 bar, respectively. Air entrainment of biodiesels was, on average, lower than those of diesel by approximately 7%.
(iii) When biodiesels were compared among themselves, it was observed that spray characteristics were very similar for all biodiesels under all experimental conditions.

As a result, it was found that biodiesels had poorer spray characteristics than fossil diesel. However, as the chamber pressure increased, the spray characteristics of the biodiesels approached those of the diesel, and there were few differences under 15 bar ambient pressure. It can be concluded that biodiesels can replace traditional diesel fuels without considerable differences in spray characteristics, as previously explained. When environmental considerations are taken into account, biodiesels are becoming even more important in decreasing pollutant emissions and reducing dependence on fossil fuels.

In the future, this study can be supported by performing a reactive spray study in terms of flame lift-off-length, flame angle, etc. Moreover, performance and emission analysis should be conducted to observe these fuels in a real diesel engine in which temperature effects are significant. Also, some different additives such as NO_*x*_ reducers such as metal-based additives and oxygenated additives can be studied with the biodiesels tested in this study.
